# Case Report: Serplulimab-induced thyroid dysfunction in a patient with advanced small cell lung cancer

**DOI:** 10.3389/fonc.2025.1611425

**Published:** 2025-11-04

**Authors:** Lu Lu, Lingxiang Liu, Wentong Fang, Xiaojian Liu

**Affiliations:** ^1^ Department of Pharmacy, Hai’an People’s Hospital, Nantong, China; ^2^ Department of Oncology, The First Affiliated Hospital of Nanjing Medical University, Nanjing, China; ^3^ Department of Pharmacy, The First Affiliated Hospital of Nanjing Medical University, Nanjing, China

**Keywords:** case report, immunotherapy, hyperthyroidism, hypothyroidism, adverse reaction

## Abstract

Studies have shown that the application of immune checkpoint inhibitors (ICIs) in solid tumors can easily affect the endocrine system, causing disorders of thyroid function, which are usually reversible after treatment cessation in most patients. Serplulimab is a PD-1 inhibitor and is used for treating small cell lung cancer (SCLC). Previous adverse reactions mention the impact of Serplulimab on the release and synthesis of thyroid hormones, but the occurrence of hyperthyroidism and hypothyroidism in the same patient has not ever been reported. We hereby report a 78-year-old patient with SCLC who developed severe hyperthyroidism after the second cycle of Serplulimab treatment. This condition lasted for one month and improved to the normal level, but it turned into hypothyroidism without any distinguishing clinical features after using Serplulimab again. The changes of relevant thyroid hormone levels in each cycle should be recorded and corresponding interventions should be implemented based on features and hormone levels. Moreover, it is necessary to combine the baseline characteristics to predict the possible adverse reactions of the patients in advance and conduct pharmacological monitoring as early as possible.

## Introduction

Immune checkpoint inhibitors (ICIs) are currently widely used in the treatment of lung cancer. Serplulimab, a recombinant programmed death 1 (PD-1) inhibitor independently developed in China, has been approved in recent years for use in combination with chemotherapy (etoposide plus carboplatin) as a first-line treatment for extensive-stage small cell lung cancer (1). To further improve therapeutic efficacy and prognosis quality, we are particularly concerned about immune-related adverse events after Serplulimab treatment. The endocrine-related adverse reactions caused by Serplulimab require vigilance from clinical practitioners, mainly including hyperthyroidism or hypothyroidism, pituitary inflammation, adrenal insufficiency, and hyperglycemia (2). In Cheng’s clinical trial, the Serplulimab group experienced a total of 26.2% thyroid dysfunction and 6.2% hyperglycemia (3). Also, Ning et al. introduced a 55-year-old male patient with SCLC who developed type 1 diabetes 68th week of receiving Serplulimab (4). The main practice contradiction of immune-related thyroid injury is that most patients have no obvious symptoms in the early stage, which is easy to be missed (5). Patial patients may have most common manifestations, like fatigue, anorexia, bradycardia etc. While others experience a short period of thyrotoxicosis including sweating, tremor, emaciation, palpitation etc. (6). Here we report a case of asymptomatic hyperthyroidism induced by Serplulimab and followed by hypothyroidism, which is alleviated through the alternative treatment of levothyroxine. Thyroid function should be routinely monitored before ICIs’ treatment, since adverse effects are more likely to occur in the first 3 months. By reviewing relevant domestic and international case reports of adverse reaction and guiding principles, we analyze and discuss the clinical characteristics and intervention measures of immune-related thyroid dysfunction caused by PD-1 inhibitors. Through this case, we propose a comprehensive pharmaceutical intervention for patients with asymptomatic thyroid dysfunction, aiming to provide references for better clinical management of such adverse reactions.

## Case presentation

A 78-year-old male patient, weighing 65 kg with a body surface area of 1.71 m², sought medical attention due to the presence of blood-streaked sputum upon waking without any obvious cause. On May 23, 2024, he was admitted to the Oncology Department of Jiangsu Provincial People’s Hospital following a confirmed diagnosis of SCLC (T4N2M1, Stage IV). From May 26, 2024, to November 6, 2024, the patient underwent six cycles of immunotherapy combined with chemotherapy: Serplulimab 200 mg on day 1, Etoposide 160 mg on days 1 to 2 or days 1 to 3, and Carboplatin 460 mg/400 mg on day 1. During this period, the patient experienced mild bone marrow suppression and severe thyroid dysfunction. The patient has a 27-year history of hypertension and has been regularly taking Levamlodipine Besylate, Rosuvastatin Calcium Tablets, and Clopidogrel Bisulfate Tablets. The patient worked as a carpenter with a history of occupational exposure to dust and radioactive materials and has not quit smoking or drinking. Discharge diagnosis including: Immunotherapy for malignant tumors, Maintenance chemotherapy for malignant tumors, Malignant neoplasm of the lung, Hypertension, Primary hypothyroidism.

**Figure 1:**
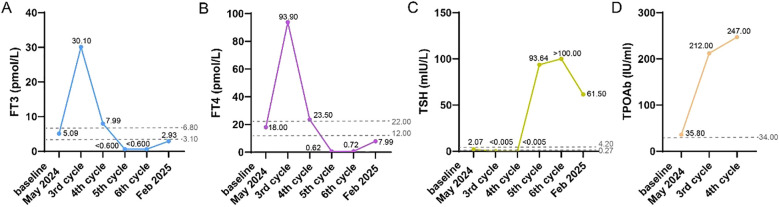
The changes of thyroid indicators from May 2024 (the baseline) to Feb 2025 (three months after the completion of 6 cycles of Serplulimab treatment), including FT3 **(A)**, FT4 **(B)**, TSH **(C)**, TPOAb **(D)**.

On November 5, 2024, the patient was admitted for the 6^th^ cycle of immunotherapy combined with chemotherapy: Serplulimab 200 mg on day 1, Etoposide 160mg on days 1–2 and 100mg on day 3, and Carboplatin 400 mg on day 1, supplemented with antiemetic and gastric-protective treatments. On July 25, 2024, the examination revealed hyperthyroidism after the 2^nd^ cycle treatment. The level of free triiodothyronine (FT3) and free thyroxine (FT4) were respectively as high as 30.10 pmol/L and 93.90 pmol/L, while thyrotropin TSH) dropped to less than 0.005 mIU/L. Meanwhile, the thyroglobulin autoantibody (TGAb) at 223.0 IU/ml and anti-thyroid peroxidase (TPOAb) at 212.0 IU/ml (the standard value of TPOAb<34 IU/ml). After receiving the fourth cycle of treatment without any intervention of the treatment plan, the patient’s FT3 and FT4 levels dropped to <0.600 pmol/L and 0.62 pmol/L, while TSH rose to 93.640 mIU/L. This condition persisted until October 5, 2024, when a subsequent hospital examination diagnosed hypothyroidism (the changes of thyroid indicators in **Figure 1**).

During the treatment, the patient remained in good condition without significant discomfort. However, persistent hypothyroidism led to abnormalities in liver and kidney function, myocardial function, and coagulation parameters after 6^th^ cycle (as detailed in the auxiliary examination results in **Table 1**). To ensure the efficacy of antitumor therapy, Levothyroxine Sodium Tablets (Euthyrox) were administered to regulate thyroid function since 5^th^ cycle. With continuous drug intervention, the laboratory indexes of each organ gradually recovered in February 2025.

**Table 1:** The biochemical and relevant parameters of baseline and after cycles of Serplulimab treatment.

Parameter	Value			Normal range
	Baseline before Serplulimab treatment (Apr 2024)	After the completion of 5 cycles of Serplulimab treatment (Nov 2024)	Three months after the completion of 6 cycles of Serplulimab treatment (Feb 2025)	
Coagulation profile				
PT (s) APTT (s) Fibrinogen (g/L) TT (s) D-dimer (mg/L)	11.70 26.90 4.29 16.00 0.41	11.80 31.50 1.91 19.3 0.4	11.20 25.20 4.21 15.6 0.45	8.0-14.0 25.0-31.3 2.0-4.0 15.0-21.0 <0.55
Cardiac markers				
CK-MB (ng/mL) Myoglobin (ng/mL) BNP (pg/mL) cTnI cTNT (ng/L)	1.40 31.20 - negative 9.85	8.75 112.4 525 negative 12.71	- - - - 9.50	0-6.22 <72 0-125 - 0-14
Complete blood count				
BA (10^9^/L) Lymphocyte (10^9^/L) RBC (10^12^/L) Platelets (10^9^/L) Hemoglobin (g/L)	0.15 1.37 4.51 263 139	0.10 1.12 3.91 167 124	0.05 1.02 4.06 122 132	0.00-0.06 1.10-3.20 4.3-5.8 125-350 130-175
Biochemical examination				
ALT (U/L) AST (U/L) Creatinine (μmol/L)	16.6 22.8 68.1	48 59 118	28 26.8 103.1	≤50.0 15-40 57-111
Tumor biomarkers				
CEA (ng/mL) CYFRA21-1 (ng/mL) NSE (ng/mL)	2.43 4.27 41.80	4.28 3.13 18.20	- - -	≤5.00 <3.30 <16.30

PT, Prothrombin time; APTT, Activated partial thromboplastin time; TT, Thrombin time; CK-MB, Creatine kinase-MB; BNP, B-type natriuretic peptide; cTnI, cardiac troponin I; cTNT, cardiac Troponin T; BA, Basophilic granulocyte; RBC, Red blood cell; ALT, Alanine aminotransferase; AST, Aspartate aminotransferase; CEA, Carcinoembryonic antigen; CYFRA21-1, Cytokeratin 19 fragment; NSE, Neuron-specific enolase.

## Discussion

The patient was treated with a regimen of Serplulimab combined with Etoposide and Carboplatin. After the second cycle of treatment, hyperthyroidism developed and persisted until August 29, 2024, during the fourth cycle of treatment. Thyroid function tests indicated that FT3, FT4, TGAb, and TPOAb levels had risen to several times the standard range, while TSH had decreased to <0.005 mIU/L. By October 2, 2024, during hospitalization, the patient transitioned to secondary hypothyroidism, which persisted until the current admission. Reviewing adverse reaction reports and multiple clinical trial results, there is no documented evidence of endocrine system disorders caused by Etoposide or Carboplatin in lung cancer patients. In contrast, Serplulimab, which mobilizes the body’s immune system to eliminate tumor cells, is more likely to cause changes in endocrine hormones, such as thyroid and adrenal function. Common endocrine-related adverse reactions associated with Serplulimab include: very common hypothyroidism; common hyperthyroidism and thyroiditis; and rare other thyroid disorders, goiter, hypophysitis, and adrenal-related diseases (7, 8). *Chinese Expert Consensus on the Emergency Management of Major Endocrine Adverse Reactions Induced by ICIs* states that patients who receive ICIs combination treatment may not show any sensible symptoms of thyroid dysfunction at low incidence, but instead exhibit a brief period of thyrotoxicosis (being a surge in the release of FT3 and FT4) (9). Two to twelve weeks later, hypothyroidism occurred, which was similar to the natural course of thyroiditis. The ASTRUM-505 randomized clinical trial compared the impact of Serplulimab versus placebo combined with chemotherapy on the survival of small cell lung cancer patients (3). The Serplulimab group demonstrated a longer median overall survival (15.4 months vs. 10.9 months). However, the Serplulimab group also had a significantly higher incidence of hypothyroidism (14.9% vs. 5%) and hyperthyroidism (11.3% vs. 3.1%) of any severity compared to the placebo group. Although Serplulimab demonstrated significant antitumor efficacy in the course of this patient’s treatment, the incidence of endocrine system dysfunction it caused was second only to that of hematologic toxicity. In this case, the patient received Serplulimab for six times. Using the WHO-UMC causality assessment method, the relationship was judged as “possible,” while the APS scoring method yielded a score of 7, indicating a causality level of “probable.”

In a retrospective data study by Iwamoto et al. (10), it was shown that patients who developed thyroid dysfunction following ICI treatment had significantly higher survival rates. The greater the degree of thyroid dysfunction, the higher the remission rate of ICI treatment. The potential pathophysiological reason for thyroid dysfunction induced by immunotherapy is believed to be immune-mediated acute inflammation, followed by destruction of the thyroid gland (11). Serplulimab treatment directly activates T cells, inducing autoimmune side effects, or alters the expression of human leukocyte antigen-isotype (HLA-DR), indirectly increasing T cell activation (12). Among patients receiving Serplulimab, hyperthyroidism typically occurs earlier, with a median onset time of approximately 1.77 months and a duration of 1.54 months. In contrast, the median time to the onset of hypothyroidism is 3.65 months (7, 8), which explains why the patient’s initial endocrine abnormality manifested as hyperthyroidism. The median half-life of Serplulimab after the first dose and at steady state is approximately 19.0 days and 24.4 days, respectively. After discontinuing the medication, the median time to the resolution of hypothyroidism is 1.87 months. When this patient developed endocrine system disorders, the clear duration far exceeded the 3- to 4-week cycle of antitumor treatment, resulting in continuous thyroid dysfunction. On the other hand, discontinuing immunotherapy due to non-grade 4 severe thyroid dysfunction would significantly impact the patient’s progression-free survival.

Prior to initiating antitumor therapy on May 25, 2024, the patient’s thyroid function indicators were within normal ranges, with no evidence of primary hyperthyroidism or hypothyroidism. However, it is noteworthy that the baseline level of TPOAb was elevated at 35.8 IU/mL, exceeding the normal range. A search on the PubMed database using the keywords “immunotherapy” and “hypothyroidism” revealed that immune checkpoint inhibitor (ICI)-induced hypothyroidism is, to some extent, predictable. A study involving 53 non-small cell lung cancer (NSCLC) patients treated with Nivolumab measured TPOAb and TGAb levels to predict the likelihood of hypothyroidism. The study found that among the 9 patients with baseline TPOAb positivity, 44% (4 out of 9) developed ICI-related hypothyroidism (13). In this case, the patient experienced the less common progression from hyperthyroidism to hypothyroidism without intervention. A possible contributing factor was the elevated baseline levels of TGAb and TPOAb, which became high-risk factors for thyroid dysfunction after the initiation of Serplulimab treatment (14, 15).

According to the *Chinese Expert Consensus on the Diagnosis and Treatment of Thyroid Diseases in the Elderly (2021 Edition)*, drug-induced hyperthyroidism or abnormalities are relatively common. For endocrine diseases of grade 3 or lower severity caused by immunotherapy, treatment may be paused or symptom-relieving medications may be administered based on the safety and tolerance of individual patients. In our case, we followed the guideline of chronic-phase over-treatment, only clinical symptoms and hormones were monitored and no measures were required. If there have other organic changes or TSH>10 mIU/L, levothyroxine sodium is first-line recommended and the initial dose is from 12.5 to 50 μg/d. The initial dose should not be excessive, and the dosage should be adjusted gradually. For patients aged 70 or older, or those with arrhythmias or osteoporosis, serum TSH levels are generally controlled within 4–7 mIU/L (6). The patient takes levothyroxine sodium 50 μg/d continuously since Oct 2024 to reduce other complications caused by endocrine-related adverse reactions. In addition, TPOAb status should be considered when TSH is between 5 to 10 mIU/L. Long-term over-replacement therapy leading to iatrogenic hyperthyroidism can easily cause atrial fibrillation, osteoporosis, sarcopenia, and frailty, necessitating regular monitoring and evaluation (16).

## Conclusion

Immune-related thyroid dysfunction is a relatively common adverse reaction following ICI treatment. However, for elderly patients, it can have secondary effects on organs such as the heart, liver, and kidneys, and severe conditions may hinder the progress of antitumor therapy. Clinicians and pharmacists should fully recognize the adverse reactions and their timing related to the endocrine system caused by ICI use. They should promptly monitor relevant signs in cancer patients and pay attention to potentially overlooked pathological manifestations, such as surface discomfort or fatigue, during the course of the disease. We focus on changes in thyroid hormones after receiving chemotherapy plus Serplulimab and changes in TPOAb level associated with autoimmune attack in this study. Unfortunately, relevant FT3, FT4 and TSH measures were not recorded in the course during 2^nd^ cycle. The patient’s general condition had improved, and he was discharged with instructions to continue taking Euthyrox as prescribed. Oral medication adherence was ensured with regular phone follow-up and outpatient service. It suggests that the antitumor treatment plan should be adjusted individually based on the patient’s physical strength and constitution, when patients are highly sensitive to medications. Additionally, for patients with baseline positivity for thyroid-related autoantibodies, as in this case, may become potential risk factors for immune-related reactions in the future. Early intervention or preventive measures should be implemented before treatment to avoid further progression of adverse reactions.

## Data Availability

The raw data supporting the conclusions of this article will be made available by the authors, without undue reservation.
